# Tuber intake is independently associated with reduced risk of Hashimoto’s thyroiditis: a community-based cross-sectional study

**DOI:** 10.3389/fendo.2026.1890093

**Published:** 2026-07-14

**Authors:** Xiaochen Ji, Guohua Hao, Bowei Su, Bing Wang, Zhengnan Gao

**Affiliations:** 1 Department of Endocrinology, the Second Affiliated Hospital of Dalian Medical University, Dalian, China; 2 Graduate School, Dalian Medical University, Dalian, China; 3 Department of Endocrinology, Affiliated Zhongshan Hospital of Dalian University, Dalian, China; 4 Department of Endocrinology, Central Hospital of Dalian University of Technology, Dalian, China

**Keywords:** autoimmune thyroid disease, carotenoids, cross-sectional study, dietary patterns, Hashimoto’s thyroiditis, Mendelian randomization, tuber intake

## Abstract

**Background:**

Hashimoto’s thyroiditis (HT) is the leading cause of autoimmune hypothyroidism, and dietary factors have been implicated in its pathogenesis through immune and metabolic pathways. We aimed to identify dietary and metabolic factors associated with HT risk in a large community cohort and to evaluate whether tuber-associated metabolites causally influence HT susceptibility using Mendelian randomization (MR).

**Methods:**

Cross-sectional analysis of 7,878 community-dwelling adults (≥40 years) from the REACTION cohort, Dalian, China. HT was defined by thyroid autoantibody seropositivity (anti-TPO ≥5.61 IU/mL or anti-Tg ≥4.11 IU/mL). Forty-nine variables spanning dietary intake, lifestyle behaviours, anthropometrics, and metabolic parameters were screened by univariable and multivariable logistic regression. For MR, eight tuber-associated metabolites identified via the FoodB database were analysed as instrumental variable exposures against a published HT genome-wide association study (GWAS; N = 395,640; 15,654 cases) using inverse-variance weighted (IVW) as the primary method, with MR-Egger and weighted median as sensitivity analyses, and MR-PRESSO to assess and correct for horizontal pleiotropy.

**Results:**

HT prevalence was 29.3% (2,305/7,878). Female sex (OR 1.97, 95% CI 1.68 to 2.33), elevated total cholesterol (OR 1.13, 95% CI 1.02 to 1.26), and lower fasting glucose (OR 0.95, 95% CI 0.91 to 1.00) were independent metabolic predictors. Adequate tuber intake (50–100 g/day; OR 0.75, 95% CI 0.64 to 0.88; *P* = 0.001) and adequate vegetable intake (OR 0.87, 95% CI 0.79 to 0.96; *P*= 0.006) were independently protective, with tuber protection most pronounced in women (OR 0.76, 95% CI 0.63 to 0.90; *P* = 0.002). A dose–response analysis revealed a U-shaped pattern: adequate intake was protective (OR 0.77) while excessive intake (>100 g/day) was associated with increased risk (OR 1.50; *P* for trend = 0.45, non-significant linear trend). Four sensitivity analyses consistently confirmed the primary findings. In univariable MR, folic acid supplementation propensity was the only metabolite with adequate genetic instrumentation (mean F-statistic = 46.75; 736 harmonised SNPs). Genetically predicted folic acid supplement use causally increased HT risk across all three MR methods (IVW: β = 0.105, SE = 0.001; *P* < 0.001; Weighted Median: β = 0.112; *P* < 0.001), with outlier-robust analysis confirming a consistent estimate after removal of 74 influential SNPs (β = 0.109; effect change 4.1%).

**Conclusions:**

Adequate tuber intake is an independent, novel protective dietary factor for HT, with a U-shaped dose–response pattern confirmed across four sensitivity analyses. Mendelian randomization identifies folic acid supplementation as a causal risk factor for HT, a finding with direct public health relevance given widespread supplement use in women of reproductive age. These findings support moderate tuber and vegetable consumption as a low-cost HT prevention strategy, and warrant caution regarding high-dose folic acid supplementation in HT-susceptible individuals.

## Introduction

Hashimoto’s thyroiditis (HT) is the leading cause of hypothyroidism in iodine-sufficient areas worldwide, accounting for approximately 20–30% of all thyroid disorders (1). Its incidence has increased in recent decades, with epidemiological evidence implicating environmental and lifestyle factors alongside genetic predisposition (1, 2). The immunopathogenesis of HT involves aberrant Th17/Treg balance and NF-κB-driven inflammation — pathways mechanistically regulated by plant-food-derived bioactives including carotenoids, dietary fibre, and polyphenols (3–8). Genetic predisposition is a non-modifiable contributor to HT development, whereas lifestyle, nutritional factors, and oxidative stress may represent meaningful areas for intervention (2).

Even for the most extensively studied micronutrients, such as selenium and vitamin D supplementation, evidence remains inconsistent, and their ability to reduce autoantibody levels varies considerably across studies (9–12). Furthermore, recent studies have examined the relationship between broader nutritional factors — including antioxidant nutrients, retinol (13)— and HT, as well as broader dietary patterns such as the Mediterranean diet (14), with findings suggesting that adequate intake of antioxidant-rich foods may represent a meaningful adjunctive strategy in disease management. This underscores the need for larger, well-characterised studies examining novel dietary exposures in relation to HT risk.

By contrast, starchy tubers — encompassing potatoes, sweet potatoes, yams, taro, and cassava, a primary plant-based energy source for billions globally — have never been systematically examined in relation to HT. Tubers are nutritionally distinctive: orange- and yellow-fleshed varieties are among the richest dietary sources of provitamin A carotenoids (α- and β-carotene); all tubers contain abundant resistant starch, a potent prebiotic; and purple-fleshed varieties are concentrated sources of anthocyanins and polyphenols (15). Dioscorea yams additionally contain dioscin, a steroidal saponin with documented immunomodulatory properties in experimental autoimmune thyroiditis models (16).

A further gap is the causal status of dietary associations with HT. Observational studies are inherently susceptible to confounding and reverse causation: subclinical thyroid dysfunction may alter dietary behaviour before clinical diagnosis. Mendelian randomization (MR) — which uses germline genetic variants as instrumental variables — provides a natural experiment largely free of these biases and is increasingly applied in nutritional epidemiology (17).

Therefore, this study addressed two questions: (1) Which dietary and metabolic factors are independently associated with HT? (2) Do tuber-derived metabolites causally influence HT risk, as assessed by two-sample MR?

## Methods

### Study population and design

**Figure 1:**
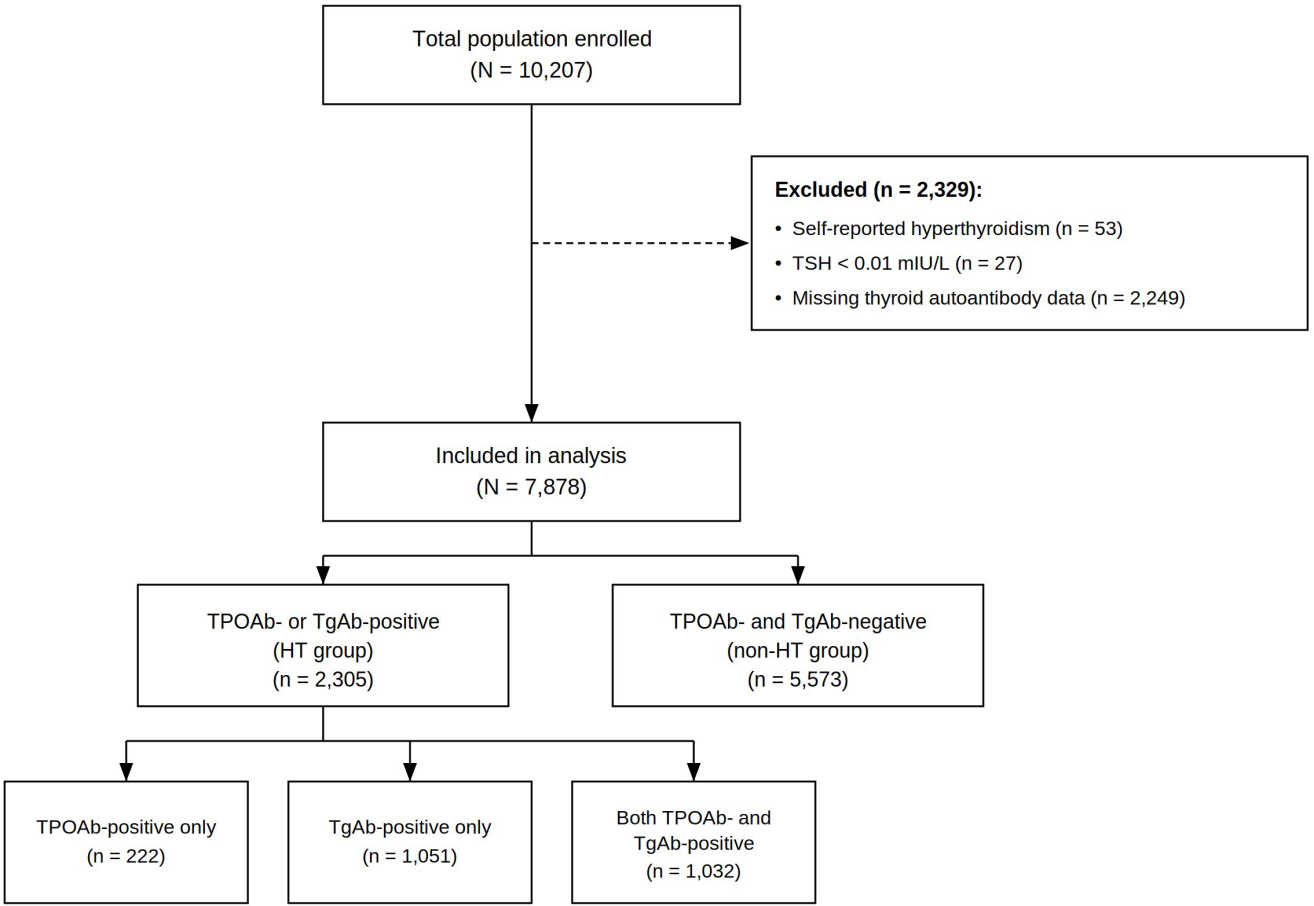
Study flowchart showing participant selection and exclusions(N = 7,878).

This cross-sectional study used data from the Dalian subcohort of the REACTION (Risk Evaluation of cAncers in Chinese diabeTic Individuals: a lONgitudinal) study (18), a multicenter community-based cohort recruiting adults aged ≥40 years from selected community health centres across multiple cities in China; in the Dalian subcohort, participants were enrolled from three community health centres using a convenience sampling approach. Of 10,207 enrolled participants, we excluded those with self-reported hyperthyroidism (n = 53), TSH < 0.01 mIU/L (n = 27), or missing thyroid autoantibody data (n = 2,249), leaving 7,878 participants for analysis (**Figure 1**). Ethical approval was granted by the Ethics Committee of Ruijin Hospital, Shanghai Jiao Tong University School of Medicine (approval no (2011). clinical ethics review, NO.14). All participants provided written informed consent prior to enrolment. All procedures were performed in accordance with the Declaration of Helsinki.

### Outcome definition

HT was defined by seropositivity for at least one thyroid autoantibody: anti-thyroid peroxidase antibody (anti-TPO) ≥5.61 IU/mL or anti-thyroglobulin antibody (anti-Tg) ≥4.11 IU/mL, measured by chemiluminescence immunoassay (Abbott ARCHITECT i2000SR). This serological definition is consistent with established community screening criteria and has been widely used in large epidemiological studies (1).

### Dietary assessment

Dietary intake was assessed using a validated semi-quantitative food frequency questionnaire (SQFFQ) covering the preceding 12 months. The questionnaire covered the following food categories: cereals, tubers, pork, beef and mutton, poultry, aquatic products, fresh vegetables, fresh fruits, soy products, dairy products, eggs, and fried foods; for each food, participants reported whether it was consumed, the consumption frequency (times per day, week, month, or year), and the amount per occasion, recorded in 50g. The intake frequency of selected items (pickled and fermented foods, coffee, and bread) and the use of nutritional supplements were also recorded. Intake of each food group was categorised using a three-level scoring system benchmarked against the Chinese Dietary Guidelines 2022 (CDG-2022) (19): score 0 = insufficient, score 1 = adequate (within CDG-2022 recommended range), score 2 = excessive. For tubers (potatoes, sweet potatoes, yams, taro, cassava and their processed forms), adequate intake was defined as 50–100 g per day. For the primary analysis, the tuber score was dichotomised: adequate (score = 1) versus non-adequate (score = 0 or 2). All other food groups were similarly dichotomised as exposure variables in logistic regression.

### Covariate assessment

Covariates included: Body mass index (BMI), calculated from baseline height (m^2^) and weight (kg) measured by trained personnel; waist-hip ratio (WHR); fasting lipid panel [total cholesterol (TC), HDL-C, LDL-C, triglycerides (TG)]; fasting plasma glucose (FPG) and 2-hour post-load glucose (2hPG) by 75 g oral glucose tolerance test; glycated haemoglobin (HbA1c); fasting insulin; serum creatinine; uric acid; liver enzymes (ALT, GGT); blood pressure (mean of three seated readings); Thyroid-stimulating hormone (TSH), free triiodothyronine (FT3), free thyroxine (FT4) — measured and reported in **Table 1** but excluded from the primary multivariable model; see Statistical analysis; thyroid ultrasound parameters (volume, isthmus thickness, gland echogenicity, nodule status — reported in **Table 1** as reference only); physical activity (International Physical Activity Questionnaire framework); sleep quality; emotional state [simplified Patient Health Questionnaire-9 (PHQ-9)]; current smoking status; and current alcohol use. All blood biomarkers were measured under fasting conditions, with the exception of 2-hour post-load glucose (2hPG).

**Table 1:** Baseline characteristics by HT status (n = 7,878).

Variable	HT group	Non-HT group	P value
Demographics			
Female sex, n (%)	1,926 (82.9)	3,807 (68.2)	<0.001
Age, years	57.8 ± 8.3	57.8 ± 8.3	0.612
Age, years (male subgroup)	61.33 ± 8.53	56.64 ± 8.49	<0.001
BMI, kg/m²	25.3 ± 3.5	25.4 ± 3.6	0.724
Waist circumference, cm	81.2 ± 9.6	82.1 ± 9.8	0.016
Thyroid function (reference only; not entered in primary model)			
TSH, mIU/L	2.41 (1.63-3.62)	1.89 (1.35-2.70)	<0.001
FT3, pmol/L	4.26 (3.98-4.51)	4.28 (4.03-4.56)	<0.001
FT4, pmol/L	13.12 (12.16-14.06)	13.23 (12.32-14.15)	<0.001
Metabolic parameters			
Total cholesterol, mmol/L	5.58 ± 1.1	5.46 ± 1.03	<0.001
LDL-C, mmol/L	3.36 ± 0.9	3.28 ± 0.87	<0.001
HDL-C, mmol/L	1.44 ± 0.33	1.41 ± 0.33	<0.001
Fasting glucose, mmol/L	6 ± 1.55	6.11 ± 1.83	<0.001
Uric acid, µmol/L	294.5 (253-343)	300 (259-351)	0.001
Creatinine, µmol/L	63 (57.9-69.5)	64.5 (58.6-72.1)	<0.001
GGT, U/L	21.5 (16.0-33.0)	23 (17.0-36.0)	<0.001
Thyroid volume, mL (women only)	13.11 ± 2.62	12.93 ± 2.1	0.032
Dietary intake			
Adequate tuber intake, n (%)†	265 (11.4)	782 (14.0)	<0.001
Adequate vegetable intake, n (%)	1228 (52.9)	3115 (55.8)	0.016
Adequate red meat intake, n (%) (male subgroup)	153 (51.7)	797 (58.0)	0.048
Lifestyle behaviours			
Current smoker, n (%)	149 (8.0)	683 (15.0)	<0.001
Current drinker, n (%)	372 (20.0)	1279 (28.1)	<0.001

HT group: n = 2,305; non-HT group: n = 5,573. Continuous variables reported as mean (SD) or median (IQR) as appropriate. Categorical variables reported as n (%). Test: t, independent-samples t-test; z, standardised Mann-Whitney z-statistic; chi2, chi-square test; chi2 (3-cat), chi-square test across three intake categories. Statistic: corresponding t, z, or chi-square value. Subgroup analyses (male subgroup, women only) noted in the variable name.

^†^Adequate tuber intake defined as 50–100 g/day per Chinese Dietary Guidelines 2022. BMI, body mass index; FT3, free triiodothyronine; FT4, free thyroxine; HDL-C, high-density lipoprotein cholesterol; GGT, gamma-glutamyl transferase (U/L); HT, Hashimoto thyroiditis; IQR, interquartile range; LDL-C, low-density lipoprotein cholesterol; SD, standard deviation; TSH, thyroid-stimulating hormone; WHR, waist-hip ratio.

### Statistical analysis

Continuous variables are summarised as mean ± standard deviation (SD) or median [interquartile range (IQR)] and compared between HT and non-HT groups by Welch’s t-test or Mann–Whitney U test as appropriate; categorical variables by chi-square test. All 49 candidate variables were first examined by univariable logistic regression; those with P < 0.05 were candidates for the multivariable model. The primary multivariable logistic regression model included all significant univariable predictors and adjusted for: sex, TC, HDL-C, LDL-C, GGT, FPG, 2hPG, uric acid, creatinine, WHR, current smoking, and current alcohol use.

Thyroid function parameters (TSH, FT3, FT4) were excluded from the primary multivariable model as, in a cross-sectional setting, these variables may reflect downstream consequences of HT-mediated thyroid destruction rather than antecedent exposures; their inclusion would risk over-adjustment bias by conditioning on a potential mediator (20). A pre-specified sensitivity analysis including TSH and FT4 is presented in **Supplementary Table 1**. Sex-stratified multivariable analyses were pre-specified given the marked female predominance of HT. Missing data (range 0.08–21.6%) were handled by random-forest iterative imputation (20 trees, 5 iterations); variables with >40% missingness were excluded. Collinearity was assessed by variance inflation factor (VIF); maximum VIF was 5.26, below the threshold of 10. All analyses were conducted in Python 3.10 (statsmodels 0.14). Statistical significance was set at two-sided P < 0.05.

As a continuous-exposure dose–response analysis, daily tuber intake (g/day) was reconstructed from the food frequency questionnaire (consumption frequency × amount per occasion; 1 *liang* = 50 g) in participants with available continuous intake records, and its association with HT risk was modelled by multivariable logistic regression with restricted cubic splines (RCS; four knots at the 5th, 35th, 65th, and 95th percentiles), adjusting for the same covariates as the primary model. Values above the 99th percentile were winsorised, and non-linearity was assessed by a likelihood-ratio test comparing the spline model with a linear-term-only model.

In a further sensitivity analysis (SA), the primary model was refitted with age and educational attainment additionally included. Although age was not statistically significant in univariable analysis, it was forced into the model given its recognised role as a risk factor for HT (1); educational attainment (three categories: primary or below, junior high school, and high school or above) was likewise included to account for potential confounding by socioeconomic position. This sensitivity analysis was performed as a complete-case analysis (**Supplementary Table 6**).

### Mendelian randomization — metabolite proxy strategy

**Figure 2:**
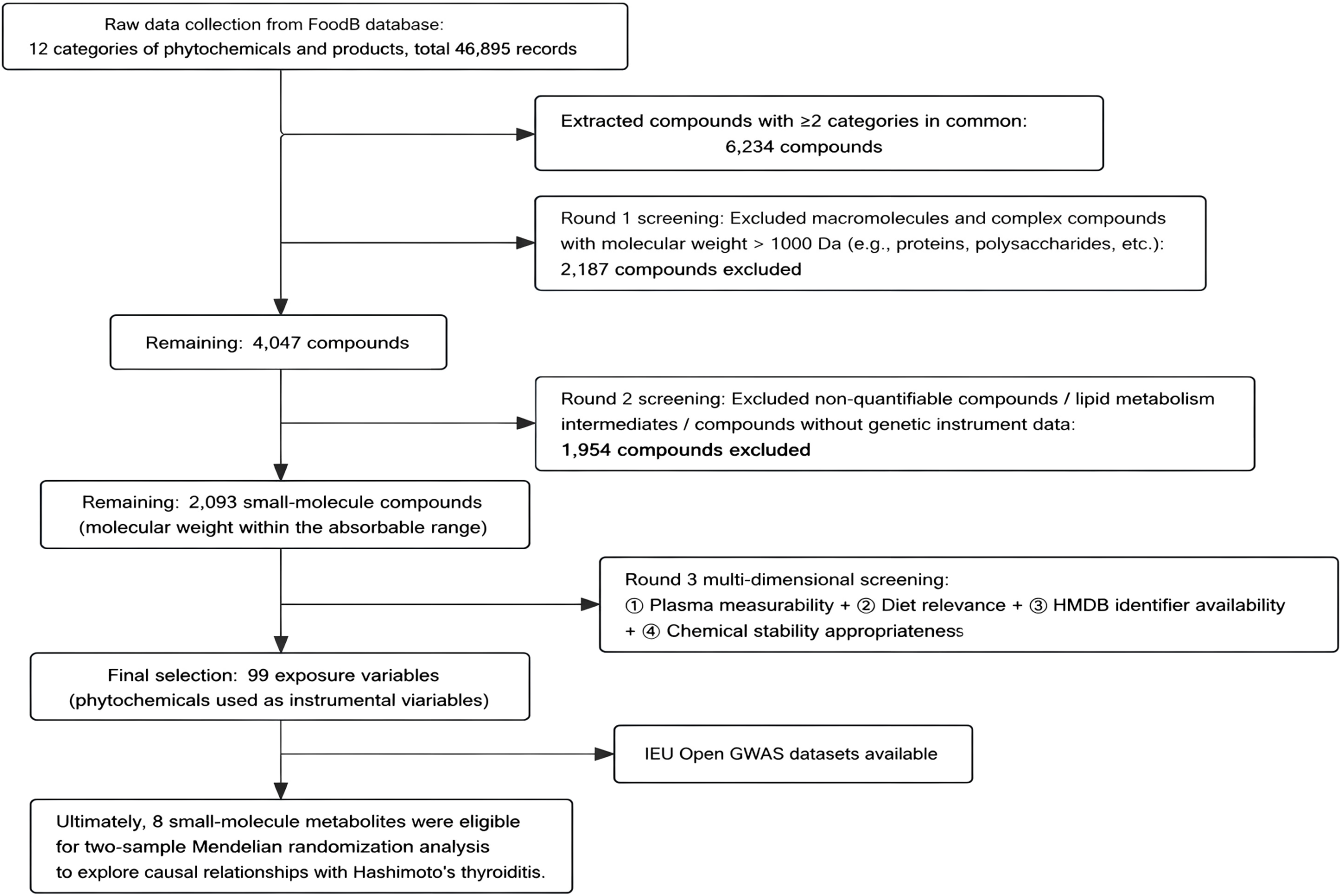
Tuber-associated metabolite screening workflow for Mendelian randomization. FoodB database entries for 12 tuber species and processing forms were sequentially filtered by shared-compound, molecular weight (<1,000 Da), bioavailability, and IEU Open GWAS availability criteria, yielding 8 final metabolite proxies for MR analysis.

**Figure 3:**
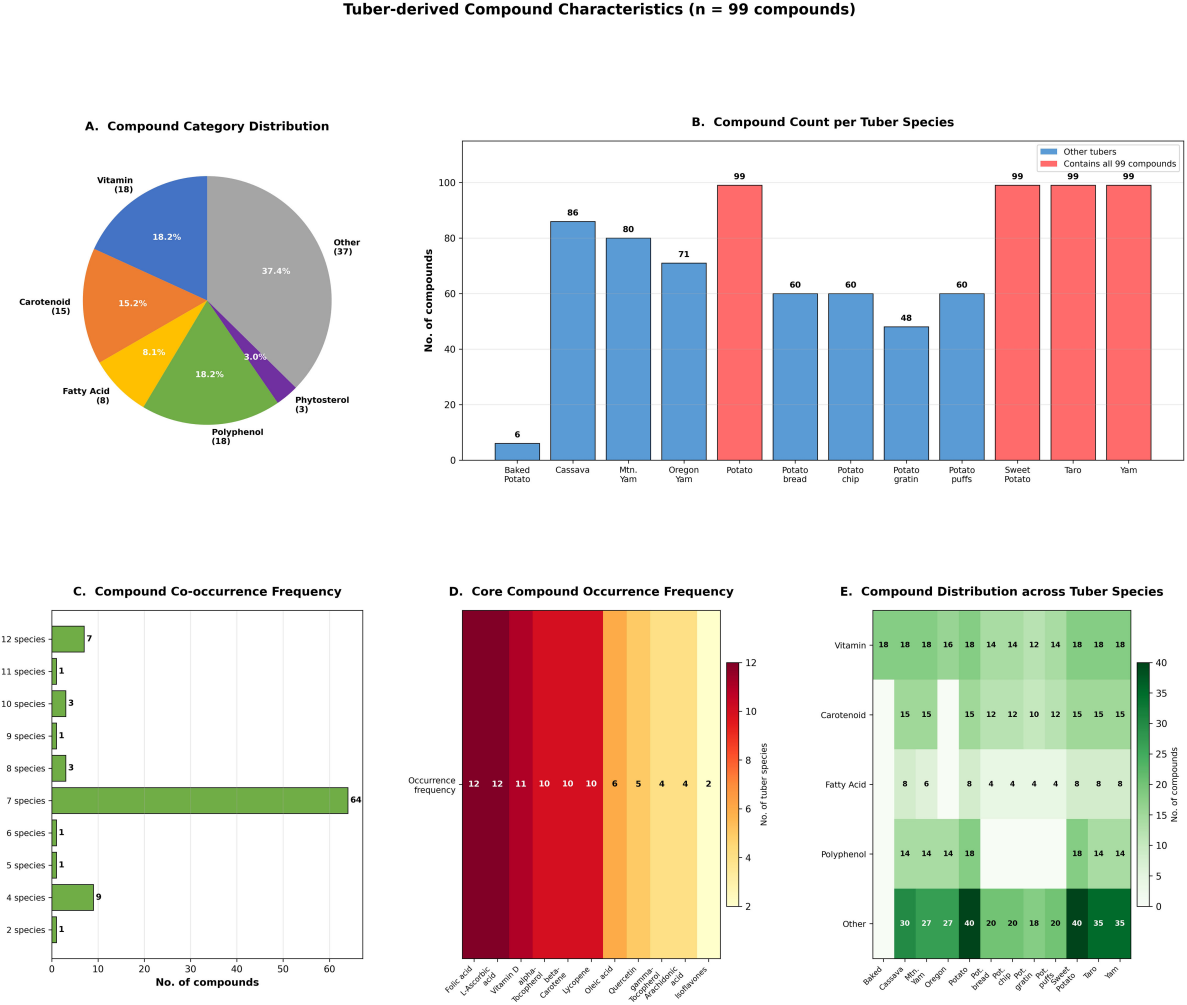
Characteristics of tuber-derived compounds identified as Mendelian randomization exposure proxies (n = 99 compounds). **(A)** Compound category distribution. Pie chart showing the proportional distribution of 99 tuber-derived compounds across six chemical categories: Vitamin (n = 18, 18.2%), Carotenoid (n = 15, 15.2%), Fatty Acid (n = 8, 8.1%), Polyphenol (n = 18, 18.2%), Phytosterol (n = 3, 3.0%), and Other (n = 37, 37.4%). **(B)** Compound count per tuber species. Bar chart displaying the number of the 99 identified compounds present in each of 12 tuber species and processed forms. Red bars indicate species containing all 99 compounds (Potato, Sweet Potato, Taro, Yam); blue bars indicate species containing a subset. **(C)** Compound co-occurrence frequency. Horizontal bar chart showing the number of compounds shared across different numbers of tuber species. The majority of compounds (n = 64) were present in exactly 7 species; 7 compounds were shared across all 12 species. **(D)** Core compound occurrence frequency. Heatmap displaying the 11 most frequently occurring compounds across tuber species, colour-coded by number of species in which each compound appears (range: 2–12). Folic acid and L-Ascorbic acid were each present in all 12 species. **(E)** Compound distribution across tuber species by category. Matrix heatmap showing the number of compounds in each chemical category (rows) present in each tuber species (columns). Colour intensity reflects compound count (range: 0–40). White cells indicate absence of compounds in that category for the respective species.

Because a genome-wide association study (GWAS) for tuber intake per se was unavailable, we employed a metabolite-proxy strategy to identify tuber-derived bioactives with available genetic instruments. The FoodB database was queried for all chemical constituents of 12 tuber species and processed forms (46,895 compound–food entries). Compounds present in ≥2 tuber species were retained; sequential filtering for molecular weight < 1,000 Da, bioavailability, and plasma detectability yielded 99 candidate small molecules. Matching to the IEU Open GWAS platform (https://gwas.mrcieu.ac.uk/) yielded 8 metabolites with adequate GWAS data for MR (**Figures 2**, **3**).

### Mendelian randomization — instrumental variable selection

Instrumental single-nucleotide polymorphisms (SNPs) were selected at genome-wide significance (*P* < 5 × 10^-8^). For exposures with no genome-wide significant SNPs, the threshold was relaxed to *P* < 1 × 10^-5^; where fewer than three instruments remained after clumping, all SNPs passing quality filters were retained and results interpreted with caution regarding potential weak instrument bias. Linkage disequilibrium clumping was applied (r² < 0.001; 10,000 kb window). Weak instruments (F-statistic < 10) were excluded. Strand-ambiguous SNPs with intermediate allele frequencies that could not be harmonised were removed.

### Mendelian randomization — analytical approach

The HT outcome GWAS (IEU Open GWAS ID: ebi-a-GCST90018855) comprised 395,640 European-ancestry participants (15,654 HT cases, 379,986 controls) (21). Primary causal effect estimates were obtained using the inverse-variance weighted (IVW) method. Sensitivity analyses employed MR-Egger regression (intercept test for directional pleiotropy) and the weighted median estimator (valid when ≥50% of instruments are valid). MR-PRESSO was applied to detect and correct for outlier-driven horizontal pleiotropy, using 10,000 permutations with Bonferroni-corrected significance threshold. Instrument strength was assessed by the mean F-statistic calculated as the average of (β_exp/SE_exp)² across all MR-ready SNPs; exposures with mean F < 10 were considered to have insufficient genetic instrumentation and their results are reported for completeness only. The Steiger directionality test was used to verify the assumed causal direction (exposure → HT). All MR analyses were conducted in Python 3.10 (numpy, scipy, statsmodels) and R 4.2.2 (MRPRESSO package).

### Patient and public involvement

Patients and members of the public were not involved in the design, conduct, or reporting of this study. Community health workers assisted with participant recruitment. Findings will be disseminated to local health authorities and the REACTION study coordinating committee.

## Results

### Participant characteristics

Of 7,878 participants, 2,305 (29.3%) met the serological definition for HT. The HT group was predominantly female (82.9% vs 68.1%, *P* < 0.001), showed significantly higher TSH (median 2.41 vs 1.89 mIU/L), total cholesterol (5.58 ± 1.10 vs 5.46 ± 1.03 mmol/L), and LDL-C, alongside lower FT3, FT4, fasting glucose, creatinine (*P* < 0.05, see **Table 1**). Rates of current smoking (6.2% vs 12.9%) and current alcohol use (4.9% vs 9.7%) were significantly lower in the HT group (both *P* < 0.001). The proportion of participants with adequate tuber intake was significantly lower in the HT group (11.4% vs 14.0%, *P* < 0.001). Adequate vegetable intake also differed significantly between groups (52.9% vs 55.8%, *P* = 0.016), as did adequate red meat intake in the male subgroup (51.7% vs 58.0%, *P* = 0.048); no other dietary food group showed a statistically significant difference. (**Table 1**; full dietary comparison in **Supplementary Table 5**).

### Univariable logistic regression

In the total population (**Table 2**), significant risk factors for HT included: female sex (OR 2.254, 95% CI 1.996 to 2.545; *P* < 0.001), total cholesterol (OR 1.116, 95% CI 1.066 to 1.168; *P*< 0.001), LDL-C (OR 1.098, 95% CI 1.039 to 1.160; *P* < 0.001), and HDL-C (OR 1.295, 95% CI 1.120 to 1.497; *P* < 0.001). Significant protective factors included: adequate tuber intake (OR 0.777, 95% CI 0.665 to 0.908; *P* = 0.002), adequate vegetable intake (OR 0.888, 95% CI 0.806 to 0.978; *P* = 0.016), fasting glucose (OR 0.942, 95% CI 0.916 to 0.969; *P* < 0.001), current smoking (OR 0.454; 95% CI 0.378 to 0.547; *P*< 0.001), and current alcohol use (OR 0.479; 95% CI 0.389 to 0.590; *P* < 0.001). In sex-stratified analyses, adequate tuber intake was protective in women (OR 0.755, 95% CI 0.633 to 0.900; *P* = 0.002), but did not reach significance in men.

**Table 2:** Univariable logistic regression: variables significantly associated with Hashimoto’s thyroiditis in the total population and gender subgroup.

Variable	OR	95% CI lower	95% CI upper	P value	Sig
Panel A. Total population (n = 7,905; HT prevalence = 29.4%; AIC = 9370.9; Pseudo-R2 = 0.024; max VIF = 5.08)					
Female sex	2.254	1.996	2.545	<0.001	***
Age, per year	1.002	0.996	1.007	0.576	ns
Total cholesterol, per mmol/L	1.116	1.066	1.168	<0.001	***
LDL-C, per mmol/L	1.098	1.039	1.160	<0.001	***
HDL-C, per mmol/L	1.295	1.120	1.497	<0.001	***
WHR	0.333	0.162	0.682	0.003	**
Adequate tuber intake †	0.777	0.665	0.908	0.002	**
Adequate vegetable intake	0.888	0.806	0.978	0.016	*
Fasting glucose, per mmol/L	0.942	0.916	0.969	<0.001	***
Creatinine, per µmol/L	0.992	0.988	0.995	<0.001	***
Uric acid, per µmol/L	0.999	0.998	1.000	0.001	***
OGTT 2h glucose, per mmol/L	0.986	0.975	0.997	0.014	*
GGT, per U/L	0.997	0.996	0.999	<0.001	***
Current smoker	0.454	0.378	0.547	<0.001	***
Current drinker	0.479	0.389	0.590	<0.001	***
Adequate red meat intake	0.926	0.839	1.022	0.127	ns
Panel B. Female subgroup (n = 5,728; HT prevalence = 33.6%; AIC = 7309.7; Pseudo-R2 = 0.004; max VIF = 5.26)					
Total cholesterol, per mmol/L	1.063	0.994	1.137	0.073	ns
LDL-C, per mmol/L	1.069	1.005	1.137	0.035	*
Adequate tuber intake †	0.755	0.633	0.900	0.002	**
Adequate vegetable intake	0.874	0.782	0.975	0.016	*
Fasting glucose, per mmol/L	0.965	0.933	0.997	0.035	*
Panel C. Male subgroup (n = 2,177; HT prevalence = 18.3%; AIC = 2079.9; Pseudo-R2 = 0.011; max VIF = 4.39)					
Age, per year	1.023	1.011	1.037	<0.001	***
Total cholesterol, per mmol/L	1.349	1.090	1.670	0.006	**
LDL-C, per mmol/L	1.110	1.000	1.232	0.049	*
Adequate tuber intake †	0.738	0.510	1.068	0.107	ns
Adequate vegetable intake	0.857	0.689	1.067	0.168	ns
Fasting glucose, per mmol/L	0.954	0.881	1.034	0.255	ns
Adequate red meat intake	0.757	0.593	0.966	0.024	*

^†^Adequate tuber intake: 50–100 g/day (Chinese Dietary Guidelines 2022). All analyses used the multiply imputed dataset (n = 7,878). CI, confidence interval; LDL-C, low-density lipoprotein cholesterol; GGT, gamma-glutamyltransferase; OGTT, oral glucose tolerance test; OR, odds ratio; WHR, waist-hip ratio; ns, not significant. ***P < 0.001; **P < 0.01; *P < 0.05; ns, *P* >= 0.05.

### Multivariable logistic regression

After full adjustment (**Table 3**), independent risk factors for HT were: female sex (OR 1.974, 95% CI 1.678 to 2.322; *P*< 0.001) and elevated total cholesterol (OR 1.120, 95% CI 1.010 to 1.243; P = 0.032). Lower fasting glucose was independently protective (OR 0.951, 95% CI 0.908 to 0.996; *P* = 0.033). Adequate tuber intake was independently protective (OR 0.754, 95% CI 0.644 to 0.884; *P* = 0.001), remaining significant after adjustment for vegetable intake, total cholesterol, and all other covariates — demonstrating a food-group-specific effect not attributable to a general healthy diet pattern. Adequate vegetable intake was also independently protective (OR 0.870, 95% CI 0.789 to 0.960; *P* = 0.006). In sex-stratified models, tuber protection was significant in women (OR 0.755, 95% CI 0.633 to 0.900; *P*= 0.002) but not in men (OR 0.738, 95% CI 0.510 to 1.068). In men, total cholesterol was the only significant independent predictor (OR 1.349, 95% CI 1.090 to 1.670; *P* = 0.006).

**Table 3:** Multivariable logistic regression: independent predictors of Hashimoto’s thyroiditis (total population and sex-stratified).

Variable	OR	95% CI lower	95% CI upper	P value	Sig
Panel A. Total population (n = 7,905; HT prevalence = 29.4%; AIC = 9370.9; Pseudo-R2 = 0.024; max VIF = 5.08)					
Female sex	1.974	1.678	2.322	<0.001	***
Adequate tuber intake†	0.754	0.644	0.884	0.001	***
Adequate vegetable intake	0.870	0.789	0.960	0.006	**
Total cholesterol, per mmol/L	1.120	1.010	1.243	0.032	*
Fasting glucose, per mmol/L	0.951	0.908	0.996	0.033	*
Current smoker	0.821	0.656	1.027	0.085	ns
Current drinker	0.875	0.687	1.116	0.283	ns
LDL-C, per mmol/L	0.957	0.851	1.075	0.456	ns
OGTT 2h glucose, per mmol/L	1.007	0.988	1.027	0.468	ns
HDL-C, per mmol/L	0.947	0.794	1.129	0.543	ns
Uric acid, per µmol/L	1.000	1.000	1.001	0.204	ns
GGT, per U/L	0.999	0.998	1.001	0.296	ns
WHR	0.872	0.413	1.841	0.719	ns
Creatinine, per µmol/L	1.000	0.997	1.003	0.953	ns
Panel B. Female subgroup (n = 5,728; HT prevalence = 33.6%; AIC = 7309.7; Pseudo-R2 = 0.004; max VIF = 5.26)					
Adequate tuber intake†	0.755	0.633	0.900	0.002	**
Adequate vegetable intake	0.874	0.782	0.975	0.016	*
Fasting glucose, per mmol/L	0.947	0.894	1.003	0.061	ns
Total cholesterol, per mmol/L	1.056	0.937	1.190	0.369	ns
Uric acid, per µmol/L	1.001	1.000	1.001	0.154	ns
GGT, per U/L	1.000	0.998	1.002	0.900	ns
Current drinker	0.685	0.361	1.302	0.249	ns
Panel C. Male subgroup (n = 2,177; HT prevalence = 18.3%; AIC = 2079.9; Pseudo-R2 = 0.011; max VIF = 4.39)					
Total cholesterol, per mmol/L	1.349	1.090	1.670	0.006	**
Current smoker	0.791	0.621	1.008	0.058	ns
LDL-C, per mmol/L	0.805	0.633	1.024	0.077	ns
GGT, per U/L	0.998	0.996	1.000	0.115	ns
Adequate tuber intake†	0.738	0.510	1.068	0.107	ns
Adequate vegetable intake	0.857	0.689	1.067	0.168	ns
Fasting glucose, per mmol/L	0.954	0.881	1.034	0.255	ns

†Adequate tuber intake: 50–100 g/day (Chinese Dietary Guidelines 2022). CI, confidence interval; GGT, gamma-glutamyltransferase; HDL-C, high-density lipoprotein cholesterol; HT, Hashimoto thyroiditis; LDL-C, low-density lipoprotein cholesterol; ns, not significant; OGTT, oral glucose tolerance test; OR, odds ratio; VIF, variance inflation factor; WHR, waist-hip ratio. ***P < 0.001; **P < 0.01; *P < 0.05; ns, *P* >= 0.05.

All variables with *P* < 0.05 in univariable analysis entered simultaneously. VIF-based collinearity screening applied (removal threshold VIF > 10). z (Wald) = Wald z-statistic (z = beta/SE). Model fit statistics and max VIF are shown in the panel headers.

### Sensitivity analyses

Four pre-specified sensitivity analyses confirmed the robustness of primary findings (**Supplementary Tables 1–4**). First (SA1), when TSH and FT4 were added to the primary model, adequate tuber intake remained independently protective (OR 0.738, 95% CI 0.627 to 0.868; *P*< 0.001). TSH was a strong independent predictor (OR 1.244, 95% CI 1.205 to 1.284; *P*< 0.001), and its inclusion attenuated but did not eliminate the tuber association, consistent with partial rather than complete mediation through thyroid function pathways. Second (SA2), restricting analysis to participants with normal TSH (0.45–4.5 mIU/L; n = 7,107) yielded a strengthened tuber OR of 0.715 (95% CI 0.599 to 0.853; *P* < 0.001), reducing the likelihood of reverse causation. Third (SA3), complete-case analysis (n = 7,793; 98.9% of the analytic sample, confirming negligible imputation impact) gave a nearly identical tuber OR of 0.760 (95% CI 0.648 to 0.891; *P* = 0.001). Fourth (SA4), dose–response analysis using the original three-category tuber score revealed a U-shaped pattern: compared with insufficient intake (score = 0; HT prevalence 29.4%), adequate intake (score = 1; HT prevalence 24.9%) was protective (OR 0.774, 95% CI 0.660 to 0.907; *P* = 0.002) while excessive intake (score = 2; HT prevalence 38.6%) was associated with higher risk (OR 1.497, 95% CI 1.187 to 1.889; *P* < 0.001). *P* for linear trend was non-significant (*P* = 0.45), confirming a non-monotonic pattern.

### Continuous dose–response analysis

**Figure 4:**
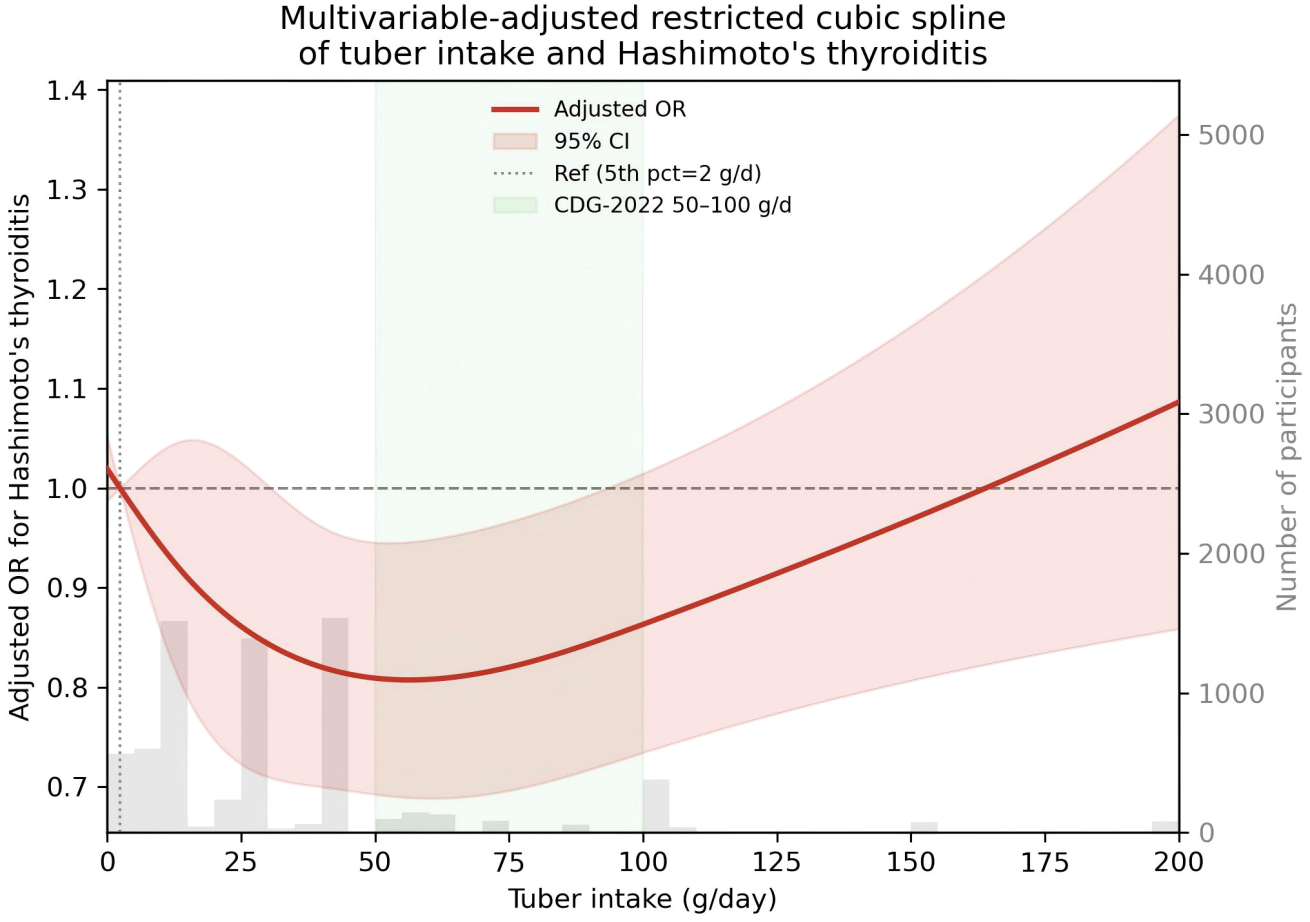
Multivariable-adjusted restricted cubic spline of tuber intake and Hashimoto’s thyroiditis. Multivariable-adjusted dose–response association between continuous daily tuber intake (g/day) and the risk of Hashimoto’s thyroiditis (n = 7,196; 2,090 cases), modelled by restricted cubic splines with four knots placed at the 5th, 35th, 65th, and 95th percentiles of intake. The model was adjusted for the same covariates as the primary multivariable model (sex, adequate vegetable intake, total cholesterol, fasting glucose, current smoking, current alcohol use, gamma-glutamyl transferase, LDL-C, 2-hour post-load glucose, HDL-C, uric acid, waist-hip ratio, and creatinine). The solid red line denotes the adjusted odds ratio (OR) and the shaded red area the 95% confidence interval. The reference point was set at the 5th percentile of intake (~2 g/day), representing minimal consumption; the OR therefore reflects risk relative to participants who consumed almost no tubers. The horizontal dashed line indicates OR = 1; the green band marks the guideline-recommended range (50–100 g/day, Chinese Dietary Guidelines 2022); the grey histogram (right axis) shows the distribution of tuber intake. Intake above the 99th percentile was winsorised and the x-axis truncated at 200 g/day for clarity. A significant non-linear, U-shaped association was observed (*P* for non-linearity = 0.004), with risk decreasing to a nadir within the recommended range and rising again at higher intake. CI, confidence interval; HDL-C, high-density lipoprotein cholesterol; LDL-C, low-density lipoprotein cholesterol; OR, odds ratio.

To complement the categorical analysis, we modelled reconstructed continuous tuber intake using multivariable-adjusted restricted cubic splines (n = 7,196; 2,090 HT cases). A significant non-linear, U-shaped association with HT risk was observed (*P* for non-linearity = 0.004). Relative to minimal intake (5th percentile, ~2 g/day), the risk of HT declined progressively to a nadir within the guideline-recommended range before rising again at higher intake (**Figure 4**). This U-shaped pattern persisted in the unadjusted model (**Supplementary Figure 6**), independently corroborating the categorical dose–response analysis (**Supplementary Figures 4, 5**).

To further address potential confounding by age and socioeconomic position, we refitted the primary multivariable model with two additional covariates forced into the model irrespective of their univariable significance: age (per year) and educational attainment (three categories: primary or below, junior high school, and high school or above; primary or below as reference)(SA6). This complete-case analysis included 6,882 participants (1,993 HT cases) with non-missing data for all covariates. The protective association of adequate tuber intake remained essentially unchanged (adjusted OR 0.745, 95% CI 0.632–0.879; *P* < 0.001), closely consistent with the primary model. Notably, although age was not significant in univariable analysis (OR 1.002; *P* = 0.479), it showed an independent association after multivariable adjustment (OR 1.011, 95% CI 1.003–1.018; *P* = 0.004), supporting its inclusion as a confounder. Educational attainment was not independently associated with HT (junior high vs primary or below: OR 1.134, *P* = 0.175; high school or above vs primary or below: OR 1.050, *P* = 0.604).

### Mendelian randomization results

Eight tuber-associated metabolites qualified as MR exposures (**Table 4**). Mean F-statistics are reported in **Table 4**. Only folic acid supplementation propensity had adequate genetic instrumentation. For this exposure, 752 SNPs were retained after LD clumping and quality control; following harmonisation with the HT outcome GWAS and removal of palindromic/non-matching variants, 736 SNPs were available for the primary MR analysis. The remaining seven metabolites had mean F-statistics of 1.0 (99.8% weak instruments); their IVW results are presented in **Table 5** for completeness but do not support reliable causal inference.

**Table 4:** Tuber-associated metabolites selected as Mendelian randomization exposure proxies.

Metabolite	GWAS source (IEU Open GWAS ID)	N	Initial SNPs	MR-ready SNPs	Notes
Folic acid	UK Biobank (ukb-b-3563)	460,000	5,917,098	752	Supplement use propensity; binary; OR interpretation
L-Ascorbic acid (Vit C)	Shin et al., 2014 (met-a-348)	2,000	2,543,891	0*	Continuous plasma; no GW-sig SNPs; all SNPs used;mean F-statistic = 1.0; reported for completeness only
Retinol (Vit A)	UK Biobank (ukb-e-100018_CSA)	1,400	9,798,678	0*	Continuous plasma; no GW-sig SNPs; all SNPs used;mean F-statistic = 1.0; reported for completeness only
Vitamin D (25[OH]D)	UK Biobank (ukb-b-18593)	417,000	8,625,788	2	Continuous plasma; 2 GW-sig SNPs; all SNPs used; mean F-statistic = 1.0; reported for completeness only
α-Tocopherol (Vit E)	UK Biobank (ebi-a-GCST90026118)	115,000	2,544,424	0*	Continuous plasma; no GW-sig SNPs; all SNPs used;mean F-statistic = 1.0; reported for completeness only
γ-Tocopherol	UK Biobank (ebi-a-GCST90026120)	115,000	2,543,768	0*	Continuous plasma; no GW-sig SNPs; all SNPs used; mean F-statistic = 1.0; reported for completeness only
Total carotene	UK Biobank (ukb-b-16202)	65,000	8,625,788	0*	Continuous plasma; no GW-sig SNPs; mean F-statistic = 1.0; insufficient genetic instrumentation — reported for completeness only
Oleic acid	Shin et al., 2014 (met-a-332)	7,800	2,544,458	2	Continuous plasma; 2 GW-sig SNPs; borderline (P = 0.051)

*For exposures with no genome-wide significant SNPs (*P*< 5 × 10^-8^), instrument selection was relaxed to *P* < 1 × 10^-5^; where <3 instruments remained, all SNPs passing quality filters were retained. HT outcome GWAS: IEU Open GWAS ID ebi-a-GCST90018855; N = 395,640 (15,654 HT cases, 379,986 controls). GW-sig, genome-wide significant; GWAS, genome-wide association study; MR-ready SNPs” denotes instruments retained after LD clumping and quality filtering, prior to harmonisation with the outcome GWAS.

^‡^Mean F-statistic calculated as the average of (β_exp/SE_exp)² across all MR-ready SNPs. Seven metabolites had mean F-statistic = 1.0 (99.8% weak instruments), indicating insufficient genetic instrumentation; only folic acid met the threshold for reliable causal inference (mean F-statistic = 46.75). Results for the remaining seven metabolites are reported for transparency only and should not be interpreted causally.

**Table 5:** Two-sample Mendelian randomization results for folic acid supplementation propensity and seven tuber-associated metabolites in relation to Hashimoto’s thyroiditis risk.

Exposure	Method	OR (95% CI)	SE	P	Mean F	Egger intercept P	Interpretation
Folic acid	IVW	OR 1.110 (1.108–1.112)	0.0009	<0.001	46.75	—	Causal risk factor
Folic acid	MR-Egger	β = 22.08 (SE = 1.307)	1.307	<0.001	—	<0.001	InSIDE assumption may be violated
Folic acid	Weighted Median	OR 1.118 (1.108–1.129)	0.0049	<0.001	—	—	Consistent with IVW
Folic acid	MR-PRESSO corrected	OR 1.115 (1.113–1.117)	0.001	<0.001	—	—	Global P<0.001; Distortion P = 0.013; effect change 4.1%
Total carotene	IVW	β = 0.0002	0.0001	0.065	1	0.079	Insufficient instruments†
α-Tocopherol	IVW	β = 0.0001	0.0001	0.138	1	0.166	Insufficient instruments†
Ascorbic acid	IVW	β = 0.0001	0.0001	0.213	1	0.241	Insufficient instruments†
γ-Tocopherol	IVW	β = 0.0001	0.0001	0.141	1	0.167	Insufficient instruments†
Oleic acid	IVW	β = 0.0002	0.0001	0.051	1	0.068	Insufficient instruments†
Retinol	IVW	β = 0.0002	0.0001	0.145	1	0.175	Insufficient instruments†
Vitamin D	IVW	β = 0.0002	0.0001	0.065	1	0.082	Insufficient instruments†

^†^Mean F-statistic = 1.0 (99.8% weak instruments); results reported for completeness only and should not be interpreted causally. MR-PRESSO Global Test P < 0.001; 74 outlier SNPs removed by Bonferroni-corrected outlier test. The IVW and Weighted Median estimates were directionally consistent (OR = 1.110 vs OR = 1.118; both P < 0.001), supporting robustness despite significant pleiotropy. MR-PRESSO outlier correction yielded OR = 1.115, representing a 4.1% change from the raw IVW estimate (Distortion Test P = 0.013). Steiger directionality testing confirmed exposure → HT for folic acid supplementation propensity. IVW, inverse-variance weighted; MR-PRESSO, Mendelian Randomization Pleiotropy RESidual Sum and Outlier test.

For folic acid supplementation propensity, genetically predicted supplement use was positively and consistently associated with HT risk across all three MR methods: IVW (β = 0.105, SE = 0.001; *P* < 0.001), Weighted Median (β = 0.112, SE = 0.005; *P* < 0.001), and MR-Egger (β = 22.08; *P* < 0.001). The MR-Egger intercept was statistically significant (intercept = 0.060, SE = 0.003; *P* < 0.001), indicating potential violation of the InSIDE assumption. MR-PRESSO confirmed significant global heterogeneity (Global Test *P* < 0.001). Following removal of 74 outlier SNPs, the outlier-corrected estimate remained directionally consistent and statistically significant (*P* < 0.001). The Distortion Test indicated a statistically significant difference between raw and corrected estimates (Distortion Coefficient = −4.17; *P* = 0.013), suggesting modest influence of outlier SNPs on effect magnitude but not on direction or significance. Steiger directionality testing confirmed the assumed causal direction (exposure → HT) for all metabolites.

## Discussion

### Principal findings

In this large community-based cohort of 7,878 adults, adequate tuber intake was identified as an independent protective dietary factor for HT — the first such report in the published literature — with the protective effect most pronounced and consistently significant among women. Four independent sensitivity analyses unanimously confirmed this finding: the association persisted after inclusion of TSH and FT4 (SA1), was strengthened in the TSH-normal subgroup with reduced reverse causation risk (SA2), was replicated in 98.9% complete-case analysis (SA3), and was part of a U-shaped dose–response pattern confirming that guideline-concordant moderate consumption is key (SA4).

Two-sample Mendelian randomization identified folic acid supplementation propensity as a causal risk factor for HT, a finding robust across three MR methods and confirmed by MR-PRESSO outlier correction. The carotenoid-mediated mechanistic pathway, while biologically plausible and supported by the observational data, could not be causally confirmed due to insufficient genetic instrumentation for circulating carotenoid concentrations in available GWAS datasets.

### Proposed mechanistic basis of tuber-associated HT protection

The most biologically plausible mechanistic link between tuber consumption and HT protection is through provitamin A carotenoids, especially abundant in orange- and yellow-fleshed tuber varieties. While formal causal inference via MR was not feasible due to insufficient genetic instruments for circulating total carotene in available GWAS datasets, the observational association is consistent with carotenoid-mediated suppression of NF-κB activation and reduction of pro-inflammatory cytokines IL-6, TNF-α, and IL-17 that characterise HT immunopathology (5, 6). This mechanistic hypothesis is further supported by observational reports of inverse correlations between circulating β-carotene and thyroid autoantibody titres (22), though causal confirmation awaits larger GWAS of circulating carotenoid concentrations.

Beyond carotenoids, tubers contribute additional mechanistic pathways that likely act synergistically. Resistant starch, abundant in tubers, is fermented by gut microbiota to butyrate and short-chain fatty acids (SCFAs) that promote Foxp3+ Treg differentiation, reinforce intestinal mucosal barrier integrity, and restore gut dysbiosis implicated in autoimmune thyroid disease (7, 8, 23). Dioscin, a steroidal saponin specific to Dioscorea yams, attenuates experimental autoimmune thyroiditis by inhibiting TLR4/NF-κB and mTOR signalling (16). Purple sweet potatoes contribute anthocyanins with additional ROS-scavenging capacity (15). This multi-pathway convergence positions the tuber food group as a composite dietary input targeting multiple immunological checkpoints simultaneously, warranting prospective investigation and mechanistic validation.

### The U-shaped dose–response pattern and public health implications

The dose–response analysis revealed a clinically important non-linear U-shaped relationship between tuber intake and HT risk. Adequate intake (50–100 g/day, consistent with CDG-2022 recommendations) was protective, while excessive intake (>100 g/day) was associated with paradoxically higher HT risk. Several mechanisms may explain the adverse association at excessive intake. Very high starchy tuber consumption imposes a high glycaemic load, potentially promoting insulin resistance and systemic inflammation. Alternatively, excessive tuber intake may reflect dietary imbalance, substituting rather than complementing other protective food groups (vegetables, legumes, lean protein). Notably, the available MR evidence does not implicate tuber-derived metabolites as causally harmful at any dose; the U-shaped pattern is more likely explained by dietary context and glycaemic load effects at excessive intake levels than by any intrinsic toxicity of tuber constituents.

For public health guidance, these findings provide additional immune-health justification for existing CDG-2022 recommendations on tuber consumption (50–100 g/day). Public health dietary education campaigns and community nutrition programmes that promote adequate — rather than simply “more” — tuber and vegetable consumption may confer dual cardiometabolic and autoimmune thyroid benefits at the population level.

### The folic acid paradox: supplemental versus dietary folate

Our MR finding that folic acid supplement use causally increases HT risk appears to contradict observational data reporting that higher dietary folate intake is inversely associated with HT (24). The resolution lies in the nature of the MR instrument: the folic acid GWAS was derived from UK Biobank responses to the question on supplement use, capturing synthetic folic acid at potentially pharmacological doses, not food-derived dietary folate. High-dose synthetic folic acid overwhelms the capacity of dihydrofolate reductase, generating unmetabolised folic acid (UMFA) in plasma, which has been shown to impair natural killer cell cytotoxicity (25) and may epigenetically alter Treg/Th17 balance through MTHFR-dependent pathways (26). Our findings therefore do not contraindicate dietary folate from food (including tubers), but suggest that high-dose folic acid supplementation warrants caution in HT-susceptible individuals, particularly those with MTHFR polymorphisms.

### Sex differences in dietary effects

The protective effect of tuber intake was significant in women but not in men, consistent with the overall female predominance of HT (82.9% of cases in this cohort). Oestrogen amplifies humoral immune responses and promotes autoantibody production, rendering women more susceptible to autoimmune diseases and potentially more responsive to anti-inflammatory dietary exposures (27, 28). Sex differences in gut microbiome composition may further interact with tuber-derived fibre and carotene metabolism to produce differential immunomodulatory effects (29). The attenuation of the tuber association in men may partly reflect reduced statistical power (male HT prevalence 18.2%; n = 2,170), as the direction and magnitude of the point estimate in men were closely comparable to those in women.

### Comparison with existing literature

To our knowledge, this is the first epidemiological or MR study to report a protective association between tuber intake and HT. Previous dietary evidence in HT has focused on Mediterranean patterns (14), gluten exclusion (30), and micronutrients (31). Studies examining individual plant food groups in relation to HT are rare, and none has previously evaluated tubers. Our observational findings are consistent with reports of inverse correlations between β-carotene and thyroid autoantibody titres (22), though formal causal evidence for this pathway awaits larger GWAS of circulating carotenoid concentrations. The folic acid finding adds to mechanistic data on adverse immunological effects of supraphysiological synthetic folate (25, 26).

### Strengths and limitations

Strengths of this study include: a large community-based sample of middle-aged and older Chinese adults (N = 7,878); comprehensive dietary, clinical, biochemical, and ultrasound characterisation; systematic MR methodology with multiple sensitivity analyses; MR-PRESSO outlier correction confirming robustness of the folic acid causal estimate, and four robustness checks including a near-complete complete-case analysis (98.9%) confirming negligible imputation artefact.

For the MR analyses, only folic acid supplementation propensity had adequate genetic instrumentation (mean F-statistic = 46.75; 736 SNPs; 0% weak instruments). The remaining seven tuber-associated metabolites, including total carotene, lacked genome-wide significant SNPs in available GWAS datasets (mean F-statistic = 1.0; 99.8% weak instruments), precluding reliable causal inference for these exposures. The carotenoid pathway therefore remains a biologically plausible but causally unconfirmed hypothesis, awaiting larger GWAS of circulating carotenoid concentrations. The MR-Egger intercept for folic acid was statistically significant and MR-PRESSO confirmed global pleiotropy (*P* < 0.001); however, three observations support overall robustness: the IVW and Weighted Median estimates were highly consistent (OR 1.110 vs 1.118); MR-PRESSO outlier correction after removing 74 influential SNPs yielded a nearly identical estimate; and the Distortion Test confirmed only a 4.1% change in effect size. The folic acid GWAS instrument captures propensity for synthetic supplement use rather than dietary folate intake, limiting biological interpretability. The HT outcome GWAS was conducted in European-ancestry populations, which may limit direct inference to the Chinese REACTION cohort. Participants were recruited from three community health centres in Dalian using a convenience sampling approach, which may limit generalisability to the broader Chinese adult population.

## Conclusions

Adequate tuber intake (50–100 g/day) is an independent, novel protective dietary factor for Hashimoto’s thyroiditis, with a U-shaped dose–response pattern confirmed across four sensitivity analyses. Mendelian randomization identifies folic acid supplementation propensity as a causal risk factor for HT. These findings suggest that adequate tuber and vegetable consumption may represent a low-cost, accessible dietary approach worthy of further investigation in the context of HT prevention, particularly in women. Prospective cohort studies and randomised trials are needed to confirm these observations and evaluate their clinical implications.

## Data Availability

The datasets presented in this study can be found in online repositories. The names of the repository/repositories and accession number(s) can be found in the article/**Supplementary Material**.

## Supplementary material

Supplementary Table 1 Sensitivity analysis SA1 — multivariable logistic regression model with TSH and FT4 included as additional covariates. Demonstrates that the protective association of adequate tuber intake persists after inclusion of thyroid function parameters (OR 0.74, 95% CI 0.63–0.87; P = 0.0002), consistent with partial rather than complete mediation.

Supplementary Table 2 Sensitivity analysis SA2 — multivariable logistic regression restricted to participants with normal TSH range (0.45–4.5 mIU/L; n = 7,107), reducing potential for reverse causation through exclusion of subclinical thyroid dysfunction.

Supplementary Table 3 Sensitivity analysis SA3 — complete case analysis without multiple imputation (n = 7,793; 98.9% of analytic sample), confirming that results are not materially influenced by the imputation strategy.

Supplementary Table 4 Sensitivity analysis SA4 — dose–response analysis using the original three-category tuber score (0 = insufficient, 1 = adequate, 2 = excessive) as dummy variables with insufficient intake as reference, confirming the U-shaped pattern (adequate: OR 0.77, P = 0.002; excessive: OR 1.50, P = 0.001; P-trend = 0.45).

Supplementary Table 5 Complete univariable logistic regression results for all 4952 candidate variables screened in the primary analysis, including all non-significant variables not shown in the main text.

Supplementary Table 6 Sensitivity analysis (SA6): multivariable logistic regression for Hashimoto's thyroiditis with additional adjustment for age and educational attainment (complete-case analysis, n = 6,882)

Supplementary Figure 1 SA4 dose–response bar chart showing HT prevalence (%) for three tuber intake levels (insufficient/adequate/excessive), illustrating the non-monotonic U-shaped pattern. Error bars represent 95% confidence intervals. P for linear trend = 0.45.

Supplementary Figure 2 Boxplots (log scale) of anti-TPO and anti-Tg antibody titers by tuber intake level (insufficient/adequate/excessive) among HT-seropositive participants (n = 2,305).

Supplementary Figure 3 Unadjusted restricted cubic spline of tuber intake and HT. Note. Unadjusted dose–response association between continuous daily tuber intake (g/day) and the risk of HT (n = 7,478; 2,176 cases), modelled by restricted cubic splines with four knots placed at the 5th, 35th, 65th, and 95th percentiles of intake, without covariate adjustment. The solid red line denotes the odds ratio (OR) and the shaded red area the 95% confidence interval, with the 5th percentile of intake (~2 g/day) as the reference, representing minimal consumption. The horizontal dashed line indicates OR = 1; the green band marks the guideline-recommended range (50–100 g/day); the grey histogram (right axis) shows the distribution of tuber intake. Intake above the 99th percentile was winsorised and the x-axis truncated at 200 g/day for clarity. A significant non-linear, U-shaped association was observed (P for non-linearity = 0.027), consistent with the multivariable-adjusted model (**Figure 4**).
