# Supplementary figures and images for: Tuber intake is independently associated with reduced risk of Hashimoto’s thyroiditis: a community-based cross-sectional study
Source: Front Endocrinol (Lausanne). 2026 Jul 14;17:1890093. doi: 10.3389/fendo.2026.1890093 (PMC13407373; doi:10.3389/fendo.2026.1890093)

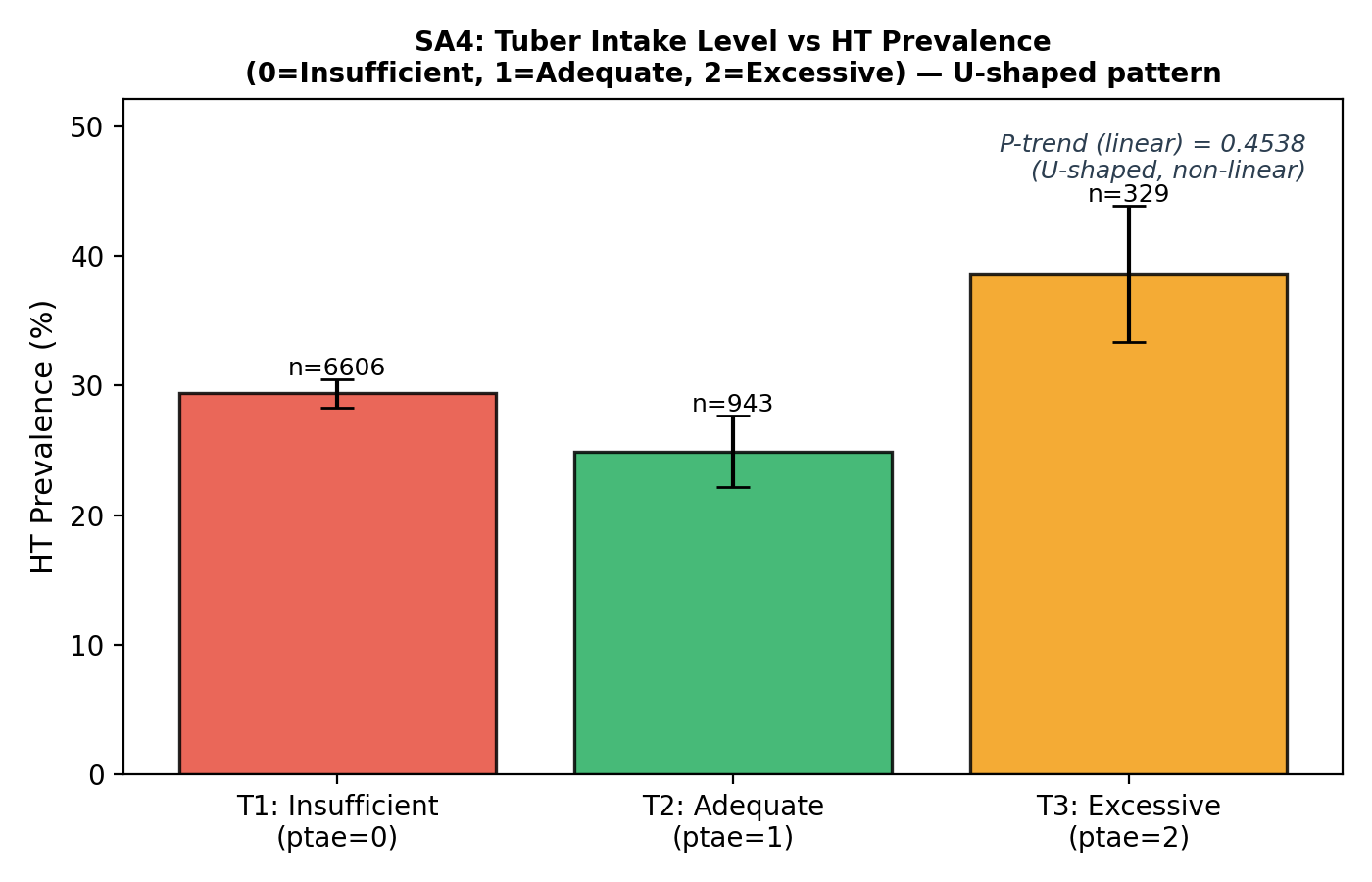

Supplement: Supplementary Figure 1 — SA4 dose–response bar chart showing HT prevalence (%) for three tuber intake levels (insufficient/adequate/excessive), illustrating the non-monotonic U-shaped pattern. Error bars represent 95% confidence intervals. P for linear trend = 0.45. [file Image1.png]

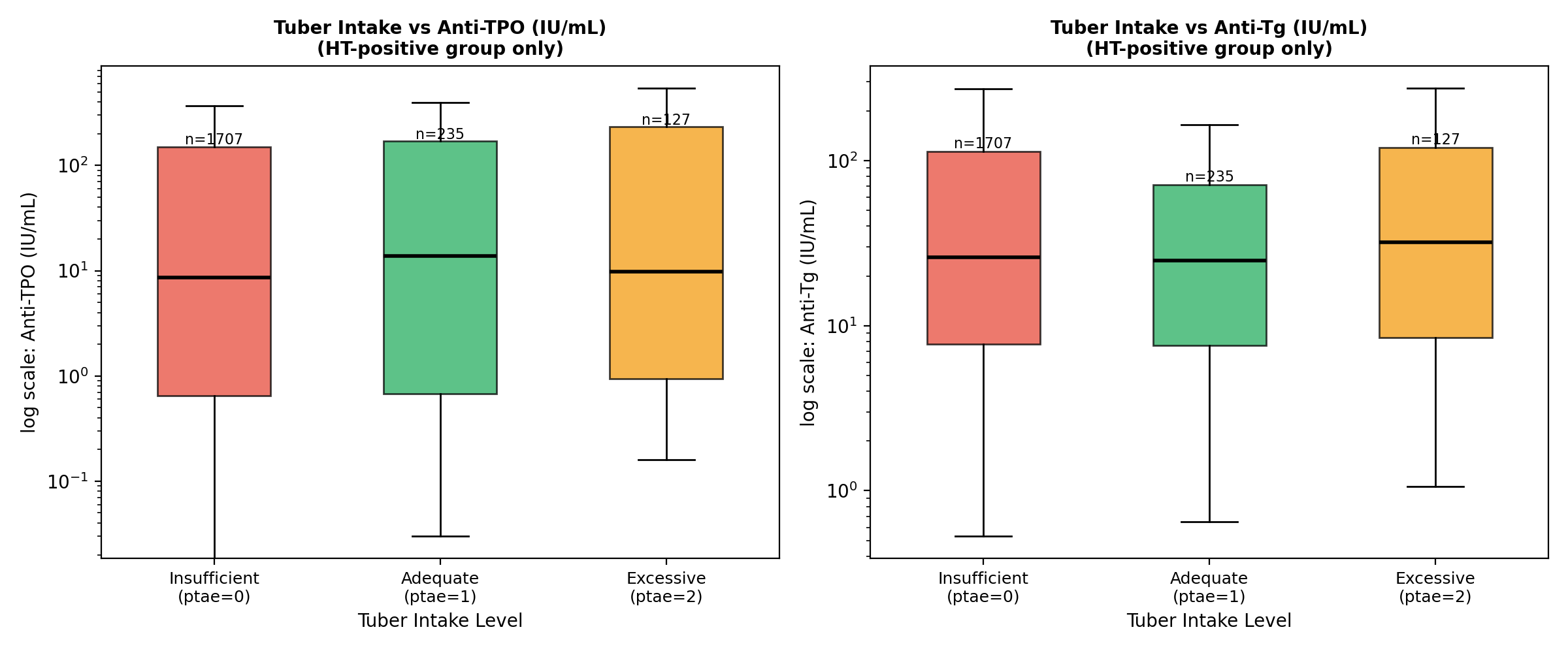

Supplement: Supplementary Figure 2 — Boxplots (log scale) of anti-TPO and anti-Tg antibody titers by tuber intake level (insufficient/adequate/excessive) among HT-seropositive participants (n = 2,305). [file Image2.png]

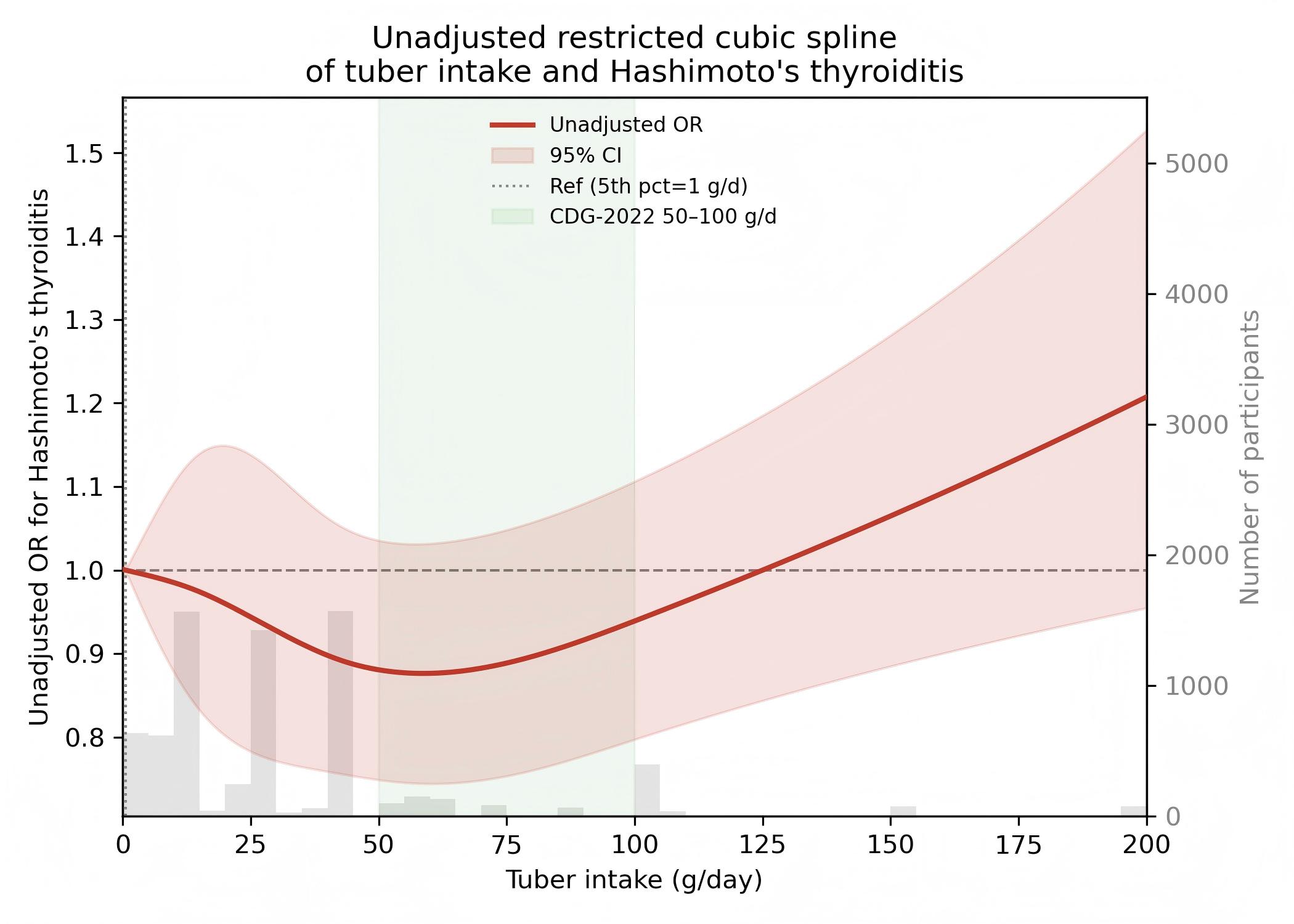

Supplement: Supplementary Figure 3 — Unadjusted restricted cubic spline of tuber intake and HT. Note. Unadjusted dose–response association between continuous daily tuber intake (g/day) and the risk of HT (n = 7,478; 2,176 cases), modelled by restricted cubic splines with four knots placed at the 5th, 35th, 65th, and 95th percentiles of intake, without covariate adjustment. The solid red line denotes the odds ratio (OR) and the shaded red area the 95% confidence interval, with the 5th percentile of intake (~2 g/day) as the reference, representing minimal consumption. The horizontal dashed line indicates OR = 1; the green band marks the guideline-recommended range (50–100 g/day); the grey histogram (right axis) shows the distribution of tuber intake. Intake above the 99th percentile was winsorised and the x-axis truncated at 200 g/day for clarity. A significant non-linear, U-shaped association was observed (P for non-linearity = 0.027), consistent with the multivariable-adjusted model (Figure 4). [file Image3.jpeg]
